# Needle-array to Plate DBD Plasma Using Sine AC and Nanosecond Pulse Excitations for Purpose of Improving Indoor Air Quality

**DOI:** 10.1038/srep25242

**Published:** 2016-04-29

**Authors:** Li Zhang, Dezheng Yang, Wenchun Wang, Sen Wang, Hao Yuan, Zilu Zhao, Chaofeng Sang, Li Jia

**Affiliations:** 1 Key Lab of Materials Modification (Dalian University of Technology), Ministry of Education, Dalian, 116024, China

## Abstract

In this study, needle-array to plate electrode configuration was employed to generate an atmospheric air diffuse discharge using both nanosecond pulse and sine AC voltage as excitation voltage for the purpose of improving indoor air quality. Different types of voltage sources and electrode configurations are employed to optimize electrical field distribution and improve discharge stability. Discharge images, electrical characteristics, optical emission spectra, and plasma gas temperatures in both sine AC discharge and nanosecond pulse discharge were compared and the discharge stability during long operating time were discussed. Compared with the discharge excited by sine AC voltage, the nanosecond pulsed discharge is more homogenous and stable, besides, the plasma gas temperature of nanosecond pulse discharge is much lower. Using packed-bed structure, where γ- Al_2_O_3_ pellets are filled in the electrode gap, has obvious efficacy in the production of homogenous discharge. Furthermore, both sine AC discharge and nanosecond pulse discharge were used for removing formaldehyde from flowing air. It shows that nanosecond pulse discharge has a significant advantage in energy cost. And the main physiochemical processes for the generation of active species and the degradation of formaldehyde were discussed.

It has been proposed that dielectric barrier discharge (DBD) can be used as a non-thermal plasma (NTP) source to improve the indoor air quality (IAQ)^1^,^2^,^3^,^4^,^5^ by removal of volatile organic compounds (VOCs), bacterial pollution, particulate matter (PM)^6^, etc. In those applications, the improvement of IAQ and the energy utilization efficiency are important parameters that should be considered emphatically. Therefore, uniform or diffuse discharge has been paid more and more attention for the advantages in obtaining a plasma with characteristics of low temperature, uniform energy distribution and moderate current density^7^,^8^. Also, the diffuse plasma with suitable gas temperature can avoid the thermal damage of catalysts, when catalysts are used to produce a synergistic effect with NTP^9^,^10^.

However, due to the instabilities of gas discharge, it is still a puzzled question to obtain a large area uniform or diffuse discharge which can remain stable for a long time in atmospheric pressure air^11^. Caused by the instabilities of gas discharge, diffuse or glow discharge can easily transfer to filament, spark, or arc discharge. Taking the typical example for glow to arc transition (GAT)^12^, there are two main reasons for GAT. Firstly, the contraction and thermalization of the discharge channel resulting from heating neutrals particles (thermal or ionization overheating instability^13^) is formed, and then the obvious heating of the cathode induced the transition of electrons emission mechanism in the discharge, which is from secondary electron emission to thermionic electron emission of electrons. Therefore, the instabilities of the discharge are always accompanied by the formation of the thermal channels such as filament, spark, or even arc. In that case, not only the energy distribution would not be optimized, but also the samples for treating, catalysts, or even electrodes can be damaged by the thermal discharge channels with high gas temperature^14^.

Therefore, one of the key issues of NTP used for improving IAQ is overcoming the limitation of discharge instability and inhomogeneity^15^,^16^,^17^,^18^, which can be achieved by using special working gas (He, Ne, N_2_ etc.), designing new electrode configurations, employing suitable excitation voltage types, and so on. As an effective method to optimize ionization efficiency^19^, nanosecond pulsed discharge (NPD), characterized by a fast rising time of pulse voltage, has unique advantages in restraining GAT and generating diffuse discharge at atmospheric pressure^20^,^21^,^22^,^23^,^24^. Since both the rising time and discharge duration of each discharge pulse are in the scale of several tens nanoseconds, electrical energy delivered in the plasma is mainly deposited in the energetic electrons instead of heating the heavy particles^21^, and also the ambient gas can sufficiently cool down after each discharge pulse^22^. Hence, NPD can be an effective method to generate stable and diffuse discharge with low gas temperature.

Besides, needle-array to plate electrode configuration (NA-P EC) can be employed to optimize electric fields and surface charge distributions. In such configuration, high density charges can be accumulated and a strong electric field can form in the region below the needle tips due to small curvature radius of the needle electrode, thus it can easily lead to a gas breakdown in the discharge gap. On the other hand, the mean population of the charges accumulated on the dielectric plates in NA-P EC is lower than that in parallel plate electrodes configuration with same projected area and electrode gap distance for the lower equivalent capacitance, so the increase in discharge current intensity can be restricted and the discharge stability can be improved effectively. In this paper, both nanosecond pulse and sine AC high voltages were used as excitation voltage to generate large area diffuse dielectric barrier discharges in NA-P EC with and without γ- Al_2_O_3_ filled in the discharge gap. Comparative studies of discharge images, electrical characteristics, optical emission spectra, and plasma gas temperatures in both sine AC dielectric barrier discharge (ACDBD) and nanosecond pulse dielectric barrier discharge (NPDBD) were carried out, and the discharge stability during long operating time were discussed. And then, both ACDBD and NPDBD in NA-P EC were used for the removal of HCHO from flowing air, and main physiochemical processes for the generation of active species and the degradation of HCHO were discussed.

## Experimental setup

**Figure 1:**
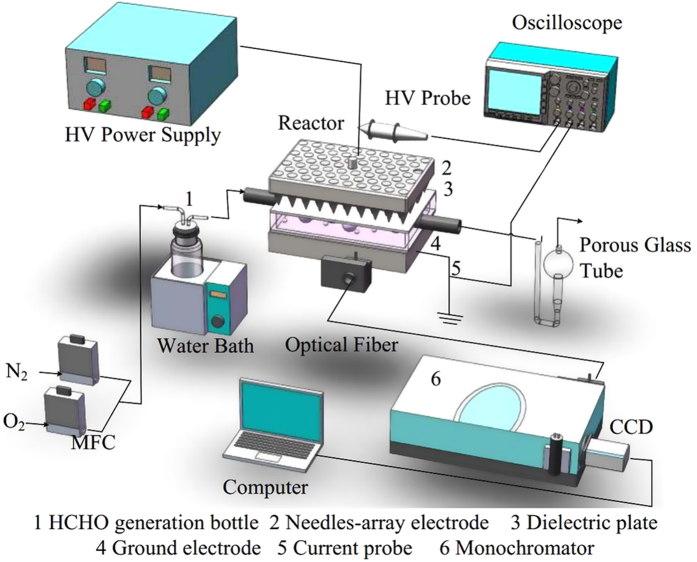
Experimental setup.

The experimental setup, shown in Fig. 1, is composed of high-voltage power supply, discharge reactor, electrical measurement system, optical detection system, HCHO generation system, and HCHO collection system. A bipolar high-voltage nanosecond pulse power supply (0–60 kV, 0–400 Hz) and a sine AC power supply (0–30 kV, 1–100 kHz) are used in the experiments. In the experiments, the applied voltage and driving frequency of ACDBD are kept at 24 kV and 9 kHz, respectively, while pulse voltage and pulse repletion rate are kept at 24 kV and 90 Hz in NPDBD, unless otherwise specified. The reactor is consisted by a needle-array electrode, two pieces of dielectric plate, a quartz frame, and a grounded electrode. The needles are made of stainless-steel with the radius of curvature of 0.4 mm and the dielectric plates are aluminum oxide ceramic plates with the thickness of 0.5 mm. The plasma is generated between two pieces of dielectric plate within the quartz frame (40 mm × 60 mm × 4 mm).

Measured with a high-voltage probe (Tektronix P6015A 1000 × 3.0 pF 75 MHz) and a current probe (Tektronix TCP312 100 MHz), the traces of discharge voltage and discharge current are recorded and displayed on an oscilloscope (TektronixTDS5054B 500 MHz). The head of optical fiber is placed close to the reactor, which can be adjustable in the vertical and horizontal directions by a 3D displacement platform. The optical emission spectra (OES) emitted from the discharge region are collected by an Andor SR-750i grating monochromator (grating groove is 2400 lines/mm with the glancing wavelength of 300 nm and grating groove is 1200 lines/mm with the glancing wavelength of 500 nm). After the diffraction of the grating, the output spectral light can be converted into a digital signal by CCD and stored in a computer.

Gas flows (N_2_/N_2_ + O_2_), controlled by mass flow controller, are premixed and bubbled into HCHO aqueous solution (concentration of 1%) in a gas washing bottle (putted into a 40 °C water bath) and then enter into the discharge reactor. Gas flows for 3 min before applying the voltage to recover the gas composition stably and discharge maintains for 3 min after applying the voltage to stabilize the treatment effect, respectively. High purity (99.999%) N_2_ and O_2_ gases are used in order to examine the effect of O_2_ on the HCHO removal efficiency. Gas flow rate is fixed at 200 ml min^−1^ and corresponding gas hourly space velocity (GHSV) is 15 625 h^−1^ in all experiments. The initial concentration of HCHO is 102 ppm. The treated gas is collected by a porous glass tube with 100 mL distilled water and the concentration of HCHO is detected by acetylacetone spectrophotometry by measuring light absorbance at 413 nm with an ultraviolet-visible absorption spectroscopy (JASCO V-550).

## Experiment results

### Diffusive characteristics from discharge images

**Figure 2:**
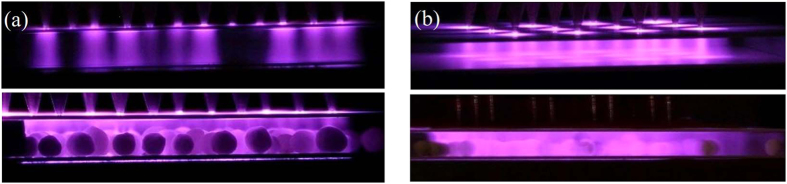
Discharge images in empty and packed bed electrode gap. (**a**) ACDBD; (**b**) NPDBD.

Fig. 2(a) shows the discharge images of ACDBD using NA-P EC in empty electrode gap and packed bed (filled with γ- Al_2_O_3_) electrode gap. The corresponding applied voltage, driving frequency and electrode gap distance are kept at 24 kV, 9 kHz and 4 mm, respectively. The exposure time of images is 20 ms, in which 180 periods of the discharges are recorded. From Fig. 2(a), it can be seen that the discharge channels are not chaotic and close to diffuse mode. Especially when γ- Al_2_O_3_ pellets are filled in the electrode gap, caused by the space charge accumulates on the surface of Al_2_O_3_ pellets, the uniformity of the discharge is improved obviously and the contraction of discharge is hardly observed by naked eyes.

Accordingly, the images of NPDBD with the pulse voltage of 24 kV and pulse repetition rate of 90 Hz are shown in Fig. 2(b). For the purpose of comparison, images of NPDBD record same discharge periods of ACDBD, which means the exposure time of NPDBD is set at 2 s. From Fig. 2(b), it can be seen that NPDBD is homogenous and can distribute into the whole electrode gap, which has an obvious different discharge mode with ACDBD. When packed bed electrode gap is used, further improvement of discharge homogeneity is obtained, and no filaments can be distinguished.

### Waveforms of voltage and discharge currents in ACDBD and NPDBD

**Figure 3:**
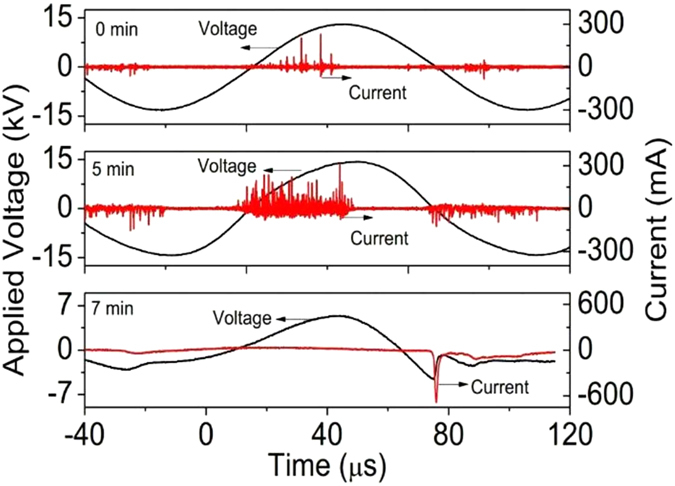
Waveforms of applied voltage and discharge current of ACDBD in different discharge duration time.

**Figure 4:**
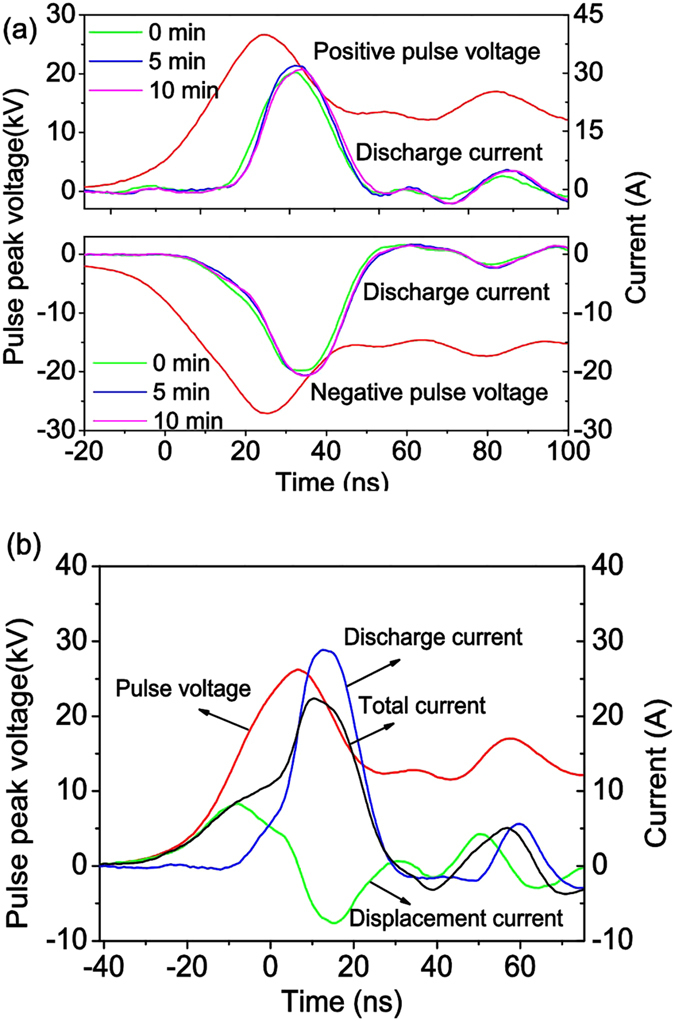
(**a**) Waveforms of applied voltage and discharge current of NPDBD in different discharge duration time; (**b**) Waveforms of pulse voltage, total current, displacement current, and discharge current in positive pulse discharge of NPDBD.

To understand the breakdown mechanisms and observe the stabilities of discharges in NA-P EC, waveforms of voltages and discharge currents in different discharge duration times (DDTs) of both ACDBD and NPDBD are recorded in Figs 3 and 4(a), respectively. Also, to indicate the discharge current in NPDBD accurately, the waveforms of pulse voltage, total current, displacement current, and discharge current in positive pulse discharge of NPDBD are shown in Fig. 4(b), in which the discharge current is obtained by detracting the displacement current from the total current detract^25^. In both Figs 3 and 4, only empty electrode gap with the distance of 4 mm is used, and both the applied voltages are kept at 26 kV in ACDBD and NPDBD.

For ACDBD, the filament characteristic can be easily observed from the waveforms of discharge current in Fig. 3, although it presents a diffusive morphology in Fig. 2(a). Evidently, at the initial phase of the discharge, DBD using NA-P EC is in a state of low amplitude of the discharge current (in the scale of 50–150 mA) and large diameter of discharge channels (up to several millimeter), which is the main distinguishing characteristic from DBD using parallel plate-plate EC, that means the current density is low enough to produce a cold plasma with moderate energy density. However, DBD excited by sine AC voltage is unstable. It is operated in a weak mode at the beginning, and then jump into an intense mode after a pre-heating time of 3–5 minutes. And when the intense discharge mode appears, the balance between the accumulation and dissipation of the input thermal energy is broken, and both the electrode and the working gas will be heated (will be discussed in *sec*. *Plasma gas temperature of ACDBD and NPDBD*). Then the transition to arc mode occurs, that means, the filament current channels are replaced by several continuous current peaks with the duration of 1–0 μs formed in the negative half period, as shown in Fig. 3. Compared with ACDBD, the waveform of discharge current in NPDBD is more regular and very stable. It is clearly shown that the discharges mainly occur at the first peaks of both positive pulse voltage and negative pulse voltage, the discharge duration time in both positive and negative pulse discharge are about 40–60 ns. The repeatability of discharge current have very good agreement for different DDTs, and both the amplitude and the peak width of discharge current are remained approximately constant.

### The effect of DDT on discharges in both ACDBD and NPDBD

**Figure 5:**
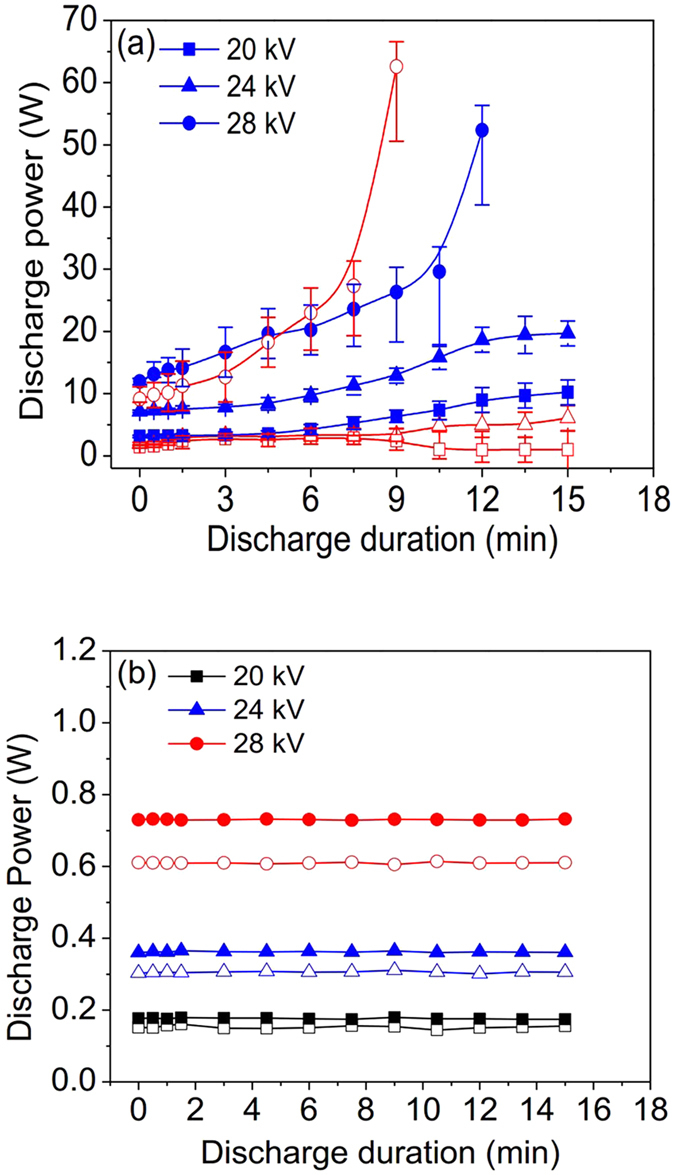
Discharge power varies as a function of DDT in the applied voltage of 20 kV, 24 kV and 28 kV. (**a**) ACDBD; (**b**) NPDBD.

Discharge stability during a long operating time is an important parameter for the applications of the NTP. To quantitatively indicate the stability of the discharge, Fig. 5(a,b) show that the discharge power of ACDBD and NPDBD varies as a function of DDT in the applied voltage (pulse peak voltage) of 20 kV, 24 kV and 28 kV. The calculation methods of discharge powers for ACDBD and NPDBD are referred in the refs 26 and 27 respectively. In the experiments, both packed bed and empty electrode gap with the distance of 4 mm are used, and they are shown with solid symbol and hollow symbol in Fig. 5, respectively. Also, the driving frequency in ACDBD and pulse repletion rate in NPDBD are kept at 9 kHz and 90 Hz, respectively.

In the case of ACDBD, when the applied voltage is 24 kV, the energy accumulation and energy dissipation in plasma can reach a balance for a long operating time. In the DDT of 0–10 min, the discharge powers of ACDBDs with both packed bed and empty electrode gap rise with the increase of DDT. And after a time (about 10–15 min) for the growing of discharge power, the discharge powers turned to be stabilized, that means the discharge powers of ACDBD with packed bed and empty electrode gap are almost constant, when the DDT is longer than 15 min. However, the range of applied voltages for stable discharge is only 4–6 kV (between 20–26 kV) in ACDBD. When the applied voltage is 28 kV, the influence of DDT on discharge power is obvious. Within 3–5 min, the discharge powers of ACDBDs increase sharply and are about 2–3 times comparing with the discharge powers at the DDT of 0 min. Then the discharge powers of ACDBDs increase continuously and a transformation to arc mode takes place at the DDT of 5–10 min. And in a relatively low applied voltage (20 kV), the effect of DDT on discharge power is indeterminate when empty electrode configuration is used. The discharge power keeps constant in the DDT of 6–10 min, then a transition of discharge mode presents. The discharge has the probability in transforming to an intense mode, or to a weak mode such as corona, or even quenched. However, the discharge mode transition cannot be observed when packed bed electrode gap is used. That is the discharge in packed bed electrode gap is more stable than the discharge in empty electrode gap.

Compared with ACDBD, the discharge power of NPDBD in both packed bed and empty electrode gap exhibits a much better stability. The discharge powers of NPDBD in Fig. 5(b) only increase with the rising of pulse peak voltage, and are almost constant with the DDT at the pulse peak voltage of 20 kV, 24 kV, and 28 kV. The large range of pulse peak voltages with long and stable operating time indicates that the NPDBD has good prospects in industry application.

### Optical emission spectra emitted from ACDBD and NPDBD

**Figure 6:**
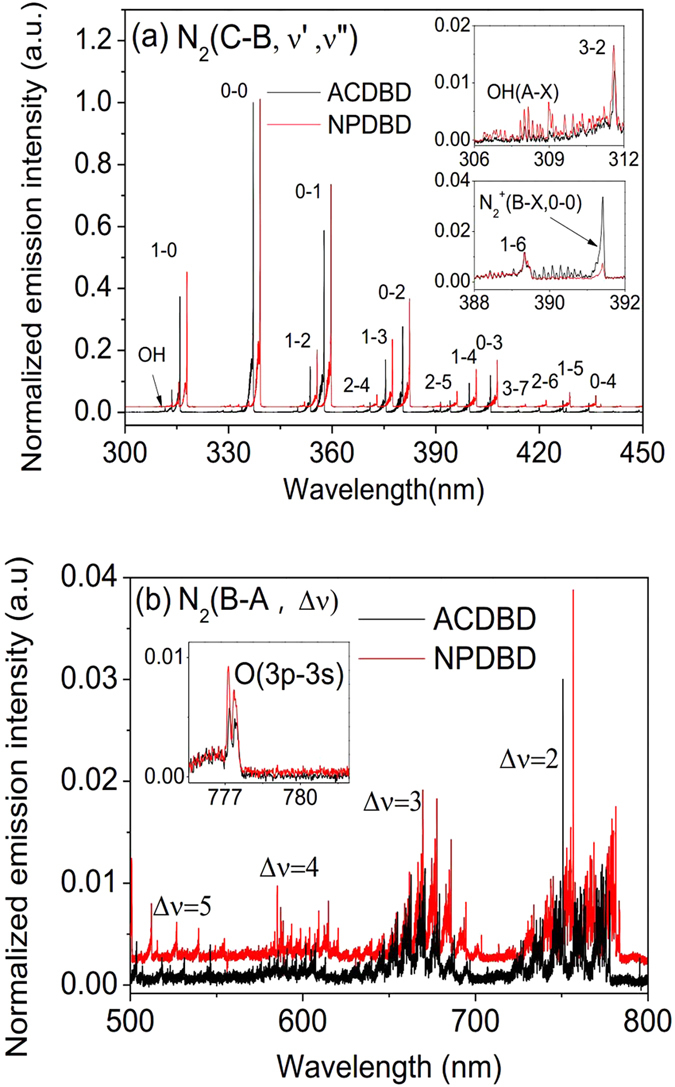
The optical emission spectra emitted from both ACDBD and NPDBD. (**a**) In range of 300–420 nm; (**b**) In range of 500–800 nm.

The optical emission spectra emitted from both ACDBD and NPDBD in range of 300–420 nm and 500–800 nm are shown Fig. 6(a,b). In order to make comparison, the spectra are normalized with the peak intensity of N_2_ (C^3^Π_u_ → B^3^Π_g_, 0-0) as 1. In the experiment, the applied voltage and driving frequency of ACDBD are kept at 24 kV and 9 kHz, correspondingly, the pulse voltage and pulse repetition rate are kept at 24 kV and 90 Hz in NPDBD. In Fig. 6, it is shown that the spectra of both ACDBD and NPDBD are mainly composed of the second positive system (SPS) of nitrogen N_2_ (C^3^Π_u_ → B^3^Π_g_) and the first positive system (FPS) of nitrogen N_2_ (B^3^Π_g_ → A^3^∑_*u*_^+^). Also weak bands of OH (A^2^Σ → X^2^Π), first negative system (FNS) of nitrogen ions N_2_^+^ (B^2^∑_*u*_^+^ → X^2^∑_*g*_^+^), and spectra lines of oxygen atoms O (3*p*^5^*P* → 3*s*^5^*S*_2_^0^, 777.4 nm) can be distinguished. The obvious distinguish of the spectra emitted from ACDBD and NPDBD are that the first negative bands N_2_^+^ (B^2^Σ_u_^+^ → X^2^Σ_g_^+^, 0-0) in NPDBD is hardly to be observed compared to that in ACDBD. According to studies of Kozlov *et al.*^28^, Massines *et al.*^29^, checking the ionic band of N_2_^+^ (B^2^Σ_u_^+^ → X^2^Σ_g_^+^, 0-0) is a safe way to judge the existing of streamer channel in the discharge. Hence, it is a powerful evidence to prove that NPDBD has a more diffusive characteristic compared with ACDBD in current conditions.

### Plasma gas temperature of ACDBD and NPDBD

**Figure 7:**
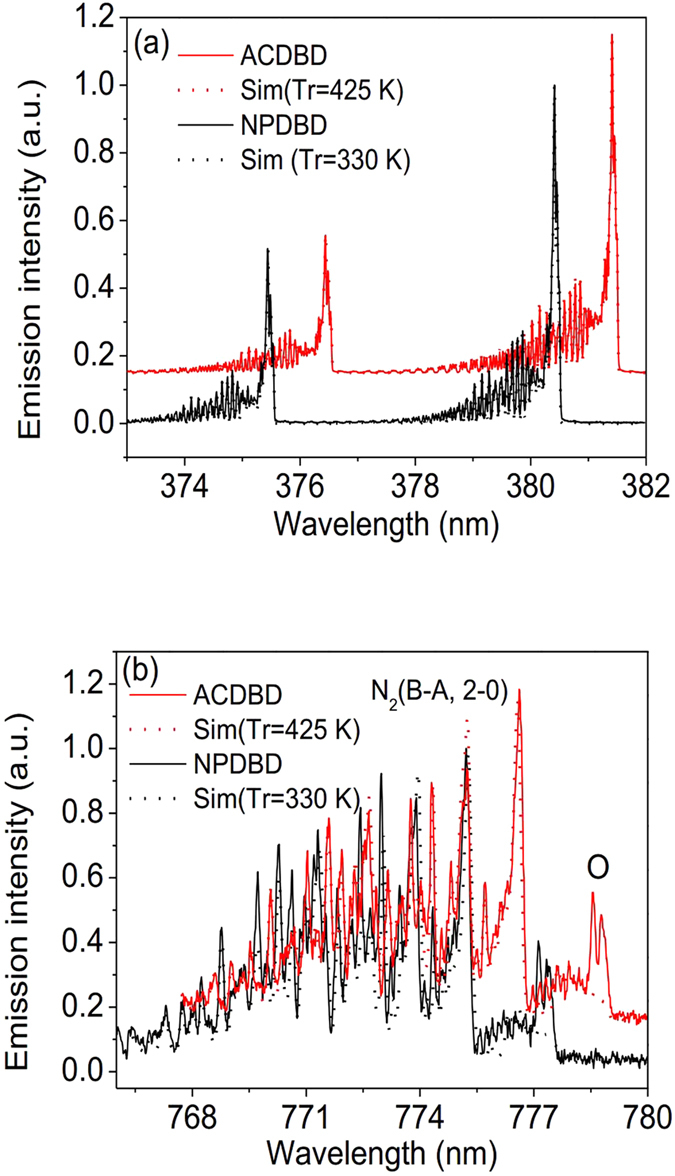
Comparing the experimental spectra and the best-fitted spectra to determine plasma gas temperature. (**a**) OES of N_2_ (C^3^Π_u_ → B^3^Π_g_, 0–2); (**b**) OES of N_2_ (B^3^Π_g_ → A^3^∑_*u*_^+^, 0–2).

Plasma gas temperature is a fundamental parameter which directly affects the plasma chemistry reaction rate and the characteristics of material surface. Because the equilibrium between translational motion and rotational motion is readily achieved by frequent collisions between the heavy particles during their radiative life at atmospheric pressure, the gas temperature is approximately equal to the rotational temperature, which can be determined by analyzing the rotational spectra of excited molecular species. In current experimental conditions, both OES of N_2_ (C^3^Π_u_ → B^3^Π_g_) and N_2_ (B^3^Π_g_ → A^3^∑_*u*_^+^) can be recorded. The N_2_ (B^3^Π_g_) state has a longer radiative lifetime than N_2_ (C^3^Π_u_), so it is a better indicator of the gas temperature. However, the bands of N_2_ (B^3^Π_g_ → A^3^∑_*u*_^+^) are very weak for distinguishing in the case of low applied voltage. Therefore, both OES of N_2_ (C^3^Π_u_ → B^3^Π_g_) and N_2_ (B^3^Π_g_ → A^3^∑_*u*_^+^) of ACDBD and NPDBD are used to calculate the gas temperature by comparing the experimental spectra and the best-fitted spectra simulated by *Specair code*^30^,^31^ and the open source code developed by Biloiu *et al.*^32^, which are shown in Fig. 7(a,b), respectively. In the experiment spectra, the applied voltage and driving frequency of ACDBD are kept 24 kV and 9 kHz, meanwhile, the pulse voltage and pulse repletion rate are kept at 24 kV and 90 Hz in NPDBD. Both the experimental spectra were recorded when the discharges have already sustained for 10 minutes, and each groups of N_2_ (C^3^Π_u_ → B^3^Π_g_, 0–2) and N_2_ (B^3^Π_g_ → A^3^∑_*u*_^+^, 0–2) are fitted at least for five times under the same conditions and error bars are given by calculating the standard deviation of the data. It can be seen that both simulated N_2_ (C^3^Π_u_ → B^3^Π_g_, 0–2) and N_2_ (B^3^Π_g_ → A^3^∑_*u*_^+^, 0–2) have good agreements with experimental spectra. In the present experimental conditions, the plasma gas temperatures of ACDBD and NPDBD are 425 K and 320 K, respectively.

**Figure 8:**
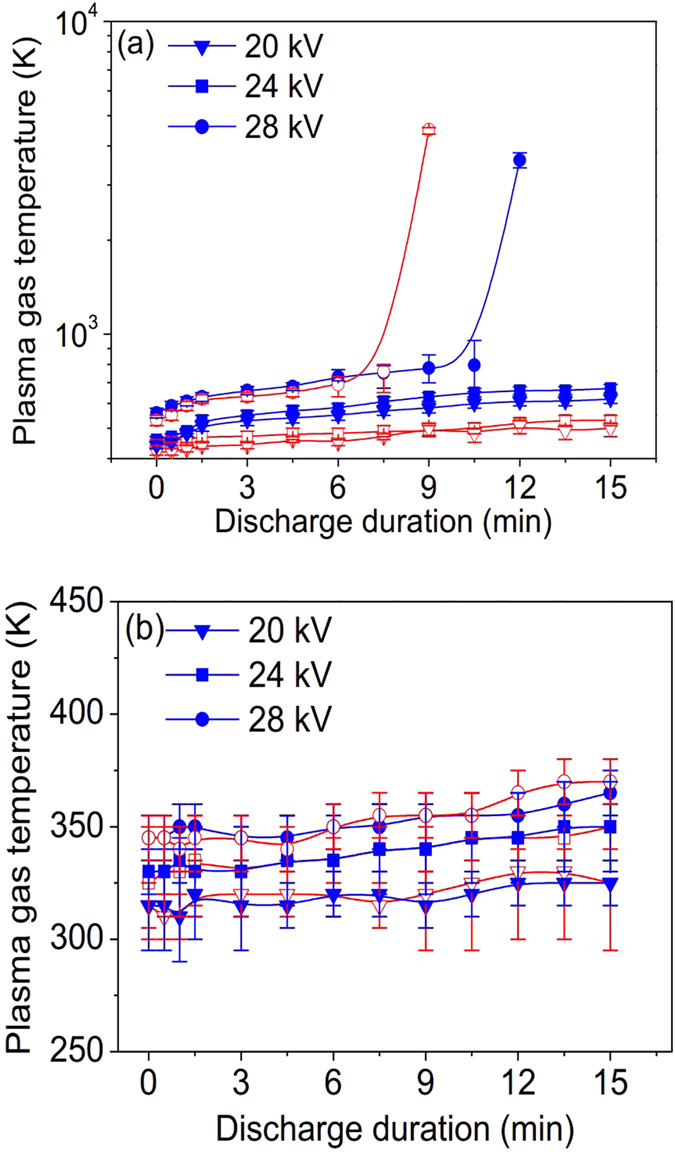
Effect of DDT on plasma gas temperatures in both empty (hollow symbol) and packed bed (solid symbol) electrode gap. (**a**) ACDBD; (**b**) NPDBD.

Plasma gas temperature is also a quantitative indicator for thermal instability of discharge. Fig. 8(a,b) show the effects of DDT on plasma gas temperatures of ACDBD and NPDBD in both empty and packed bed electrode gap under the applied voltages (pulse peak voltage) of 20 kV, 24 kV and 28 kV, respectively. In ACDBD, when the applied voltage is 20 kV, plasma gas temperature is slightly higher than environment temperature (320–350 K) for both empty and packed bed electrode gap discharges, and both of them keep almost constant with the increase of DDT. When the applied voltage is 24 kV, the gas temperature of ACDBD turns to be unstable at the beginning of the discharge, which is about 420 K at the DDT of 0 min and then rises with the increases of DDT. After 6–9 minutes, the gas temperature rises to 620–650 K and tends to be stable. However, when the applied voltage is 28 kV, the plasma gas temperature increase sharply to at least 3000–4000 K at the DDT of 3–5 min.

Compared to ACDBD, the instability of the gas temperature in NPDBD is not observed. Gas temperatures keep almost constant as the increase of DDT but increase slowly from 320 K to 350 K when the pulse peak voltage increases from 20 kV to 28 kV. The characteristics of low gas temperature and good stability during long operating time in large range of pulse peak voltages indicate that NPDBD has promising application potentials in many industrial fields, especially for improving IAQ without the damage of catalytic agent.

**Figure 9:**
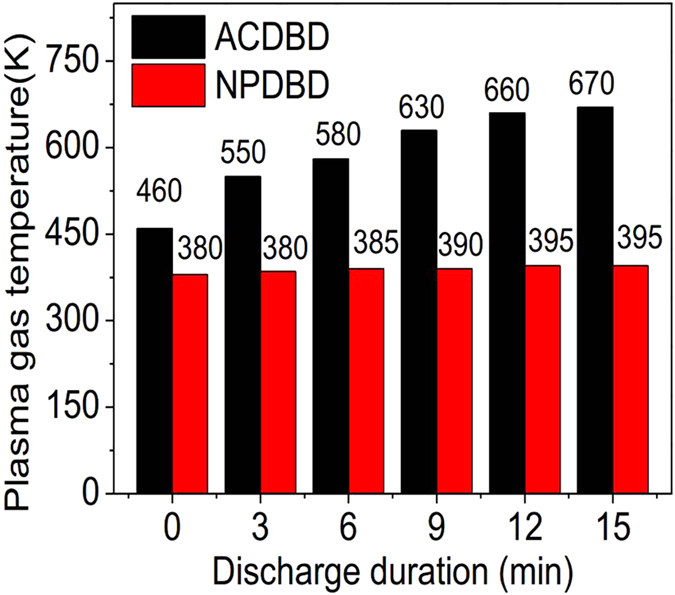
Effects of DDT on plasma gas temperatures of ACDBD and NPDBD in packed bed electrode gap with fixed initial discharge power (7.15 W).

For the gas heating, it has a direct relationship with the input discharge power. Figure 9 shows the effects of DDT on plasma gas temperatures of ACDBD and NPDBD with fixed initial discharge power in packed bed electrode gap. In the experiment, the applied voltage and drive frequency of ACDBD are 24 kV and 9 kHz, and correspondingly, the pulse peak voltage and pulse repetition rate of NPDBD are kept at 38 kV and 320 Hz for obtaining a same discharge power with ACDBD, which is about 7.15 W at the initial of the discharge. It can be seen that the plasma gas temperature of NPDBD is much lower than that of ACDBD. Although a much higher pulse peak voltage in NPDBD is introduced, the stability of NPDBD is much better than ACDBD, and the plasma gas temperature of NPDBD still keep almost constant with the increase of discharge duration time.

### ACDBD and NPDBD used for removal of HCHO

**Figure 10:**
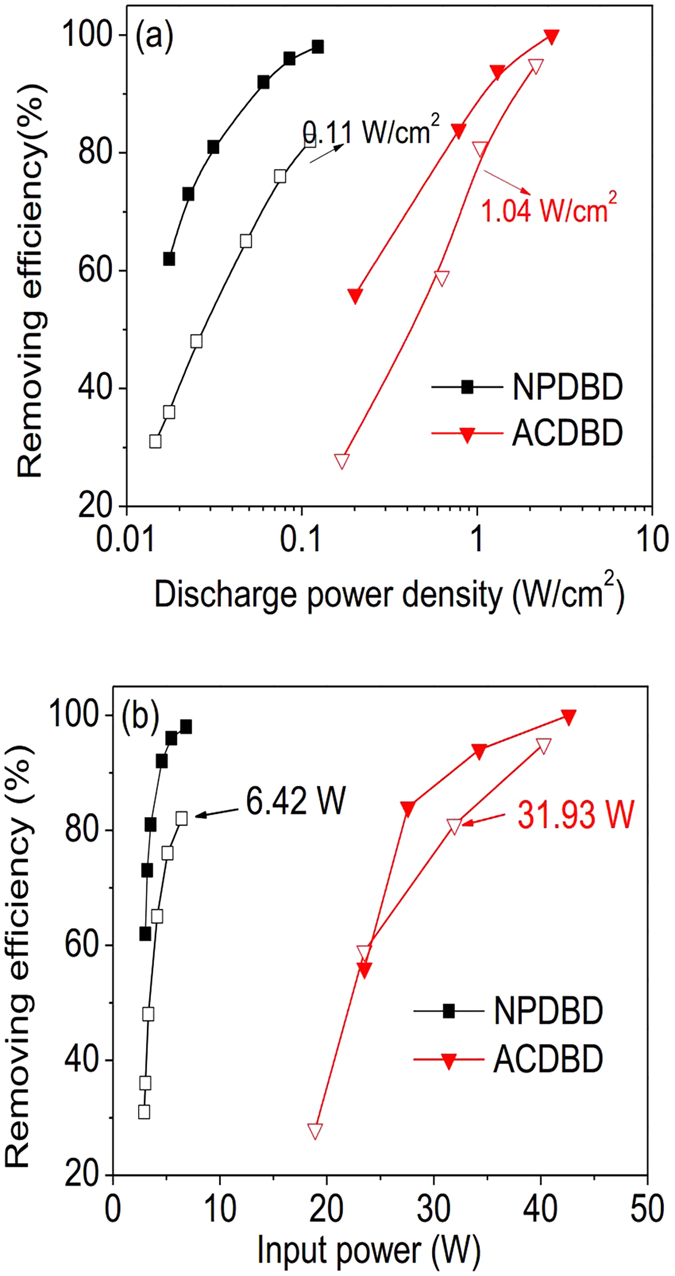
Effects of discharge power densities and input powers on the removing efficiency of HCHO in both ACDBD and NPDBD with empty (hollow symbol) and packed bed (solid symbol) electrode gap. (**a**) Effects of discharge power densities; (**b**) Effects of input powers.

HCHO is a common pollutant of IAQ, which can cause human disease including irritation of the eyes and respiratory tract, headache, thirst, etc. In this section, both ACDBD and NPDBD are employed to remove HCHO for improving IAQ. Figure 10(a) shows the effect of discharge power density on removing efficiency of HCHO in ACDBD and NPDBD. Both packed bed and empty electrode gap configurations are used and TiO_2_ as a catalyst is adsorbed on the surface of γ- Al_2_O_3_ pellets in the case of packed bed electrode gap. In the experiment, the driving frequency in ACDBD and pulse repletion rate in NPDBD are kept at 9 kHz and 90 Hz, respectively. When empty electrode gap is used, both the removing efficiencies of ACDBD and NPDBD increase with the rising of the discharge power density. A higher final removing efficiency can be obtained when ACDBD is used, which is mainly caused by the high power density and high plasma gas temperature. However, for the same removing efficiency of HCHO, NPDBD presents unique advantage in low discharge power density. To obtain a removing efficiency of about 80%, the discharge power densities of NPDBD and ACDBD are 0.12 W/cm^2^ and 6.25 W/cm^2^ respectively. That is the discharge power density of NPDBD is only about 1/50 of ACDBD. When γ- Al_2_O_3_ pellets with catalyst are filled in the electrode gap, both the removing efficiencies of HCHO in ACDBD and NPDBD increase approximately 10~30% and both removal efficiencies can reach to about 100%.

The energy consumption in the process of formaldehyde removal is an important criterion in evaluation of plasma in industrial application. Figure 10(b) shows the effects of input powers of nanosecond pulse power supply and sine AC power supply on the removing efficiency of HCHO with empty and packed bed electrode gap discharge, respectively. Although the energy efficiency of the nanosecond pulse power supply used in the experiments is lower than the sine AC power supply, the energy consumption of NPDBD is still much lower than that of ACDBD. Corresponding to the discharge power consumption, to obtain a removing efficiency of about 80%, the energy costs of NPDBD and ACDBD are 6.42 W and 31.93 W, respectively.

**Figure 11:**
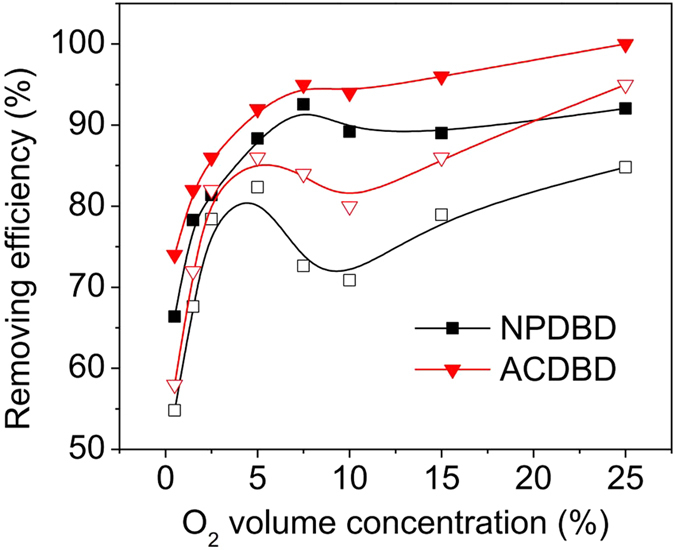
Effects of O_2_ concentrations on HCHO removing efficiencies in both ACDBD and NPDBD with empty (hollow symbol) and packed bed (solid symbol) electrode gap.

To investigate the removing mechanism of HCHO by active species, the effects of O_2_ concentration on HCHO removing efficiency in both ACDBD and NPDBD are shown in Fig. 11. Similarly, both packed bed electrode gap and empty electrode gap configurations are used. It can be seen that the concentration of oxygen has obvious influence on the removing efficiencies of HCHO. When O_2_ concentration is lower than 5%, the removing efficiencies of HCHO have a sharply increase with the increase of O_2_ concentration in all the discharge types. As further O_2_ concentration increasing, HCHO removing efficiencies exhibit a peak value when the O_2_ concentration is about 5% but decrease with the increase of O_2_ concentration, and then increase slowly with the increase of O_2_ concentration When the O_2_ concentration is larger than 15% for both ACDBD and NPDBD in empty electrode gap.

## Discussions

### Generation mechanism of the diffuse discharge in NA-P EC

Although diffuse discharges can be presented in both ACDBD and NPDBD when NA-P ECs are used, the breakdown modes of them are different. For ACDBD, the discharge mode is filament mode, as shown in Fig. 3. The diffusive morphology is mainly caused by the large diameter of discharge channel and non-vertical discharge channel distribution. In the NA-P EC, the distribution of memory charges accumulated on the surface of the ceramic plate near needle electrode is non-uniform, and the charge density in the region near needle tips is much higher than that in other regions. Therefore, electric field distribution is non-uniform and direction is non-vertical. Discharge channels originate from corona discharge near the needle tips, and then spread in both vertical and horizontal directions. In this process, discharge channels with the diameter of several millimeters can be formed by the overlap of multi-streamers. Especially when γ- Al_2_O_3_ pellets are filled in the electrode gap, caused by the charges accumulated on the surface of pellets, the direction of electric field is multifarious more ever. With the ignition of the initial corona near the electrode tips, these charges on pellets can participate in the discharge effectively, and the diffusive or homogenous characteristics can be performed in the discharge images. On the other hand, needle-array electrode has a smaller equivalent capacitance compared with the plate-plate electrode with same discharge areas, and the smaller equivalent capacitance reduced the total populations of charges accumulated on the dielectric barrier plate. Therefore, the discharge current intensity of the subsequent discharge after initial breakdown is limited, then the discharge presents a more diffusive characteristic.

In NPDBD, NA-P EC plays a same role with ACDBD. Non-uniform electric, initial corona, and smaller equivalent capacitance of NA-P EC are beneficial to the generation of diffuse discharge. Besides, for the short rising time and short pulse duration of pulse voltage in NPDBD, there are other three factors for the generation of diffuse discharge. Firstly, for the short discharge duration, the development of gas heating/ionization instability can be prevented. The ionization efficiency is optimized and the electrical energy delivered in the discharge plasma is mainly deposited in the energetic electrons instead of heating the heavy particles. So the discharge mode transition can be controlled. Secondly, the providing space charges in the discharge can be limited by the structure of double layers dielectric plates, which means the current density of the discharge is decided by the memory charges accumulated on the surface of the dielectric plate, and the developing of spark or arc can be controlled. At last, since the discharge duration in each pulse period (in scale of 10 ms) is only 40–60 ns, the duty cycle of the discharge is in the scale of 1/10^6^, and the ambient gas can be sufficiently cooled down when the next breakdown takes place, therefore, the NPDBD can present good uniformity and stability characteristics.

### Discharge stability during long operating time

The instability of the discharge during long operating time is mainly caused by the thermal processes of working gas and electrodes. In ACDBD, since the excitation voltage is continuous sine AC wave voltage, the directional movement of ions cannot be neglect, which means the working gas and electrode can be heated by positive ions and neutral particles. Then temperature of the working gas in electrode gap increases and the thermal expansion of the working gas takes place, which leads to the decrease of particle number density *N* and increase of reduce electrical field *Θ* = *E/N*, where the *E* is the electric field density.

As in the study of Shao *et al.*^21^, the electron energy in the discharge can be evaluated by the Einstein’s equation 33, as shown in equation (1),


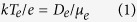


where *k*, *T*_e_, *D*_*e*_, and *μ*_e_ are Boltzmann constant, electron temperature, the electron diffusion constant and electron drift mobility. The ratio of *D*_e_ and *μ*_e_ can be expressed as a function of *E*/*N*, thus the increase of *E/N* leads to the rise of *D*_e_/*μ*_e_, which leads to the rise of electron mean energy. On the other hand, the rise of *E/N* can produce more electrons in the discharge region. Therefore, if the balance of the accumulation and dissipation of the input energy is broken, the increase of gas temperature can lead to the increase of discharge power.

Since the discharge intensity can be directly influenced by gas temperature, the stability of diffuse ACDBD mainly depends on the balance between the accumulation and dissipation of input thermal energy. In a higher applied voltage, this balance can be destroyed by the overfull thermal energy input, and the discharge intensity increases. Then a positive feedback of thermal and discharge intensity can be formed. The discharge current increases continuously, which is the reason for filament to arc transition.

For NPDBD, plasma gas temperature almost keeps constant although the pulse voltages and the DDT increase, which means gas temperature can keep close to room temperature in a large range of the pulse voltages and long operating time. For the short rising time of pulse voltage, the electrical energy is consumed to generate the energetic electrons during the discharge instead of heating plasma gas, which makes NPDBD exhibits a low gas temperature. On the other hand, in each discharge period, short discharge duration time (about 40–60 ns) and long discharge period (about 6.7 × 10^6 ^ns) guarantee that there is enough time for the plasma to cool down sufficiently, therefore the thermal instability can be prevented effectively.

### Dynamic processes of the generation of active species and the degradation of HCHO

For NTP generated by DBD, the population of excited particles can directly influence the intensity of specified spectra line or bands. And the excited particles are mainly produced by the collisions among the electrons, ions, molecules, and atoms. In HCHO–air system, the reactions and rate constants for the main physiochemical processes are listed in Table 1.

For the electron impact electronic-vibrational excitation reactions, the kinetics of the electronic states excitation, dissociation, and ionization of neutral particles are described in reactions 1–12. Since the N_2_ molecule has higher resonant-radiative states than O_2_ molecule, the excited N_2_ molecule is the dominant excited molecule in the plasma. Under current experimental condition, for the higher reaction rates and lower excitation level, the formation reactions of the energetic metastable molecule are dominant reactions, that is, reactions 1–3.

In an oxygen-rich system, the quenching of numerous metastable molecules by oxygen molecules is an important process for atomic and molecular dynamics processes, which plays an important role in gas heating and production of reactive species, such as O, OH, N. The main processes for quenching processes of N_2_ (A) and N_2_ (B) are shown in reactions 13–26 in Table 1.

Considering high reaction rate constant of reactions 13, 14, and 18, when oxygen is added in, mestable molecules N_2_ (A) can be quickly quenched by collisions with O_2_ in a short time, e.g., from reactions 13, 14, N_2_ (A) can be quenched to N_2_ (X) with the production of atoms O radicals in a time


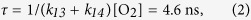


where [O_2_] is the particle number density of O_2_ which is about 5.6 × 10^19 ^cm^−3^. The fast transformation time of N_2_ (A) made the plasma particularly rich in atoms O. Compared with the rate constants for productions of N and OH, the atoms O is the dominant radical in the air discharge plasma.

The active species react with HCHO molecules and lead to the removal of HCHO. These processes are listed in reactions 27–34, in which reactions 27–29 are the initial reaction channels. In atmospheric air, the concentrations of N_2_ (A), OH, and HO_2_ etc. are very low, and reaction 28 can be considered as the main reaction channel for the degradation of HCHO. Then, the CHO generated by the degradation of HCHO can be dissociated and oxidized to CO_2_ and CO.

**Figure 12:**
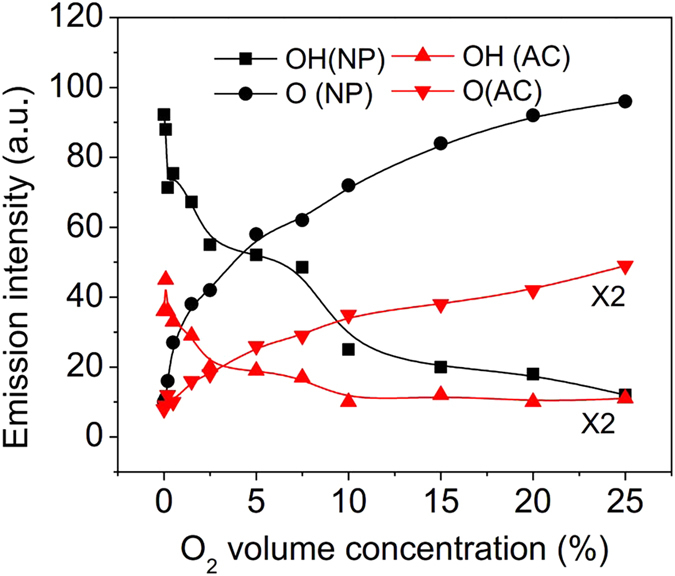
Effect of the concentration of O_2_ on the emission intensities of O (3*p*^5^*P* → 3*s*^5^*S*_2_^0^) and OH (A^2^Σ → X^2^Π, 0-0) in ACDBD and NPDBD with same energy intensity.

There are several factors that can decide the efficiency of the degradation of HCHO, such as the quenching of N_2_ (A) and OH, the adsorption of free electrons, and the production of O atoms, which are affected by the concentration of O_2_. To discuss the degradation of HCHO by O, OH, etc., the effect of the concentration of O_2_ on the emission intensities of O (3*p*^5^*P* → 3*s*^5^*S*_2_^0^) and OH (A^2^Σ → X^2^Π, 0-0) in ACDBD and NPDBD with same energy intensity are shown in Fig. 12. To obtain the same energy intensity, the exposure times of ACDBD and NPDBD are kept at 0.01 s and 2 s, respectively. In can be seen that the emission intensities of OH (A^2^Σ → X^2^Π, 0-0) in both ACDBD and NPDBD decrease sharply with the increase of O_2_ concentration. And when O_2_ concentration is higher than 5%, emission intensities OH (A^2^Σ → X^2^Π) in both ACDBD and NPDBD are close to zero. However, the emission intensities of O (3*p*^5^*P* → 3*s*^5^*S*_2_^0^) increase with the increasing of O_2_ concentration in both ACDBD and NPDBD.

When the concentration of O_2_ is low (pure nitrogen), the mestable molecule N_2_ (A) can be a dominant factor for the degradation of HCHO by reaction 32. With the increase of O_2_, the mestable molecule N_2_ (A) can be quenched by the reactions 13, 14, etc. and the main production for these reactions is O atoms. Besides, O atoms can also be generated by the dissociation process reactions 9–11. Consequently, low concentration of O_2_ has obvious influence on HCHO removing.

On the other hand, OH radicals can exist in the plasma when the concentration of O_2_ is very low. Therefore, reaction 27 with high rate constant can be an important process for the degradation of HCHO. When O_2_ concentration is higher than 5%, OH radicals can be quenched by O and O_3_ from reactions 35 and 36, thus, the influence of OH (A^2^Σ → X^2^Π) begins to neglect, and the removal efficiencies of HCHO decrease with the increasing of O_2_ concentration when the O_2_ concentration is in the range of 5%–15% in both ACDBD and NPDBD with empty electrode gap. In addition, O_2_ is a kind of electronegative gas that can capture free electrons by the reactions 37 and 38 35, so the added O_2_ can result in the reduction of free electron density greatly, which has a negative effect on HCHO removing efficiencies.

## Conclusions

In conclusion, diffuse discharges are generated in a needle-array to plate electrode configuration when both sine AC and nanosecond pulse are employed as excitation voltages. Non-uniform electric field, initial corona, and smaller equivalent capacitance formed by needle-array electrode configuration are beneficial to the generation of diffuse discharges. Waveforms of voltage and discharge current are measured to investigate the breakdown mechanisms of ACDBD and NPDBD in this electrode configuration. In ACDBD, the discharge mode is filament. Due to the non-uniform electric field, discharge channel with the diameter of several millimeters can be formed by the overlap of multi-streamers. And in NPDBD, the waveform of discharge current is much more regular, the discharge with the duration of about 40–60 ns mainly appears at the first peaks of both positive pulse voltage and negative pulse voltage. The stabilities of both ACDBD and NPDBD for long operating time are investigated in aspects of discharge power and plasma gas temperature. It is found that the NPDBD has more homogenous characteristics, better stability, and lower plasma gas temperature compared with ACDBD. Moreover, the placement of γ- Al_2_O_3_ pellets for packed bed electrode gap has obvious efficacy in the production of homogenous discharge. The optical emission spectra of ACDBD and NPDBD are used to diagnosis the excited active species and discuss the main processes for the generation of active species and the degradation of HCHO. It is found that the plasma is particularly rich in atoms O, and the reaction between O atoms and HCHO molecules can be considered as the main reaction channels for the removal of HCHO in air.

## Additional Information

**How to cite this article**: Zhang, L. *et al.* Needle-array to Plate DBD Plasma Using Sine AC and Nanosecond Pulse Excitations for Purpose of Improving Indoor Air Quality. *Sci. Rep.* **6**, 25242; doi: 10.1038/srep25242 (2016).

## Figures and Tables

**Table 1:** Reactions and rate constants for the HCHO–air system.

The excitation processes			
1	e + N_2_ → N_2_ (A) + e	log k_1_ = −8.4−14/Θ	34
2	e + N_2_ → N_2_ (B) + e	log k_2_ = −8.2−14.8/Θ	34
3	e + N_2_ → N_2_ (C) + e	log k_3_ = −8.2−21.1/Θ	34
4	e + O_2_ → O_2_ (a) + e	log k_4_ = −10.2−0.35/Θ (Θ>4)	34
5	e + O_2_ → O_2_ (b) + e	log k_5_ = −11.2−0.72/Θ (Θ>4)	34
The dissociation processes			
6	e + N_2_ → N (^4^S) + N (^2^S) + e	–	34
7	e + N_2_ → N (^4^S) + N (^2^D) + e	–	34
8	e + N_2_ → N (^4^S) + N (^2^P) + e	–	34
9	e + O_2_ → O (^3^P) + O (^3^P) + e	log *k*_*9*_ = −7.9−13.4/*Θ*	34
10	e + O_2_ → O (^3^P) + O (^1^D) + e	log *k*_*10*_ = −8−16.9/*Θ*	34
11	e + O_2_ → O (^3^P) + O (^1^S) + e	log *k*_*11*_ = −8.8−11.9/*Θ*	34
The ionization processes			
12	e + N_2_ → N_2_^+^ + e + e	–	34
The quenching processes of N_2_ (A) and N_2_ (B)			
13	N_2_ (A) + O_2_ → N_2_ (X) + O +O	*k*_*13*_ = 2.54 × 10^−12^	34
14	N_2_ (A) + O_2_ → N_2_O +O	*k*_*14*_*=*7.8 × 10^−14^	34
15	N_2_ (A) + O → NO +N (^2^D)	*k*_*15*_ = 7 × 10^−12^	34
16	N_2_ (A) + O →N_2_ (X) + O (^1^S)	*k*_*16*_ = 2.1 × 10^−111^	34
17	N_2_ (A) + NO → N_2_(X) + NO	*k*_*17*_ = 7 × 10^−11^	34
18	N_2_ (A) + O_2_ → N_2_ (X) + O_2_ (*a, b*)	*k*_*18*_ = 1.29 × 10^−12^	34
19	N_2_ (A) + O_2_^−^→ O_2_ + N_2_ + e	*k*_*19*_ = 2.1 × 10^−9^	34
20	N_2_ (A) + N_2_ (A) → N_2_ (C) + N_2_ (X)	*k*_*20*_= 2.1 × 10^−12^	34
21	N_2_ (A) + N_2_ → N_2_ (X) + N_2_	*k*_*21*_ = 3 × 10^−18^	34
22	N_2_ (A) + N (^4^S) → N_2_ (X) + N (^2^P)	*k*_*22*_ = 5 × 10^−11^	34
23	N_2_ (B) + N_2_ → N_2_ (X) + N_2_	*k*_*23*_ = 5 × 10^−11^	34
24	N_2_ (B) → N_2_ (A) + *hν*	*k*_*24*_ = 1.5 × 10^5 ^s^−1^	34
25	N_2_ (B) + NO → N_2_ (X) +NO	*k*_*25*_ = 2.4 × 10^−10^	34
26	N_2_ (B) + O_2_ → N_2_ (X) + O +O	*k*_*26*_ = 3 × 10^−10^	34
The degradation processes of HCHO			
27	HCHO + OH → CHO + H_2_O	*k*_*27*_ = 9.4 × 10^−12^	10
28	HCHO + O → CHO + OH	*k*_*28*_ = 1.7 × 10^−13^	10
29	HCHO + H → H_2_ + CO	*k*_*29*_ = 3.8 × 10^−14^*T*^1.05^ exp (−1650/*T*)	10
30	HCHO + HO_2_ → H_2_O_2_ + CHO	*k*_*30*_ = 5.0 × 10^−12^ exp (−6580/*T*)	10
31	CHO + N_2_ (A) → CO + H + N_2_ (X)	*k*_*31*_ = 5.7 × 10^−11^	10
32	CHO + OH → CO + H_2_O	*k*_*32*_ = 1.7 × 10^−10^	10
33	CHO + O_2_ → CO_2_ + OH	*k*_*33*_ = 5.0 × 10^−12^	10
34	CHO + O → CO_2_ + H	*k*_*34*_ = 5.0 × 10^−12^	10
The quenching processes of OH			
35	OH + O → H + O_2_	*k*_*35*_ = 3.8 × 10^−11^	35
36	OH + O_3_ → HO_2_ + O_2_	*k*_*36*_ = 6.5 × 10^−14^	35
The capturing processes of free electrons			
37	e + O_2_ → O_2_^−^	–	35
38	e + O_2_ + M → O_2_^−^ +M	*k*_*38*_ = 3.0 × 10^−31^	35

The rubric for this table is as follows: column 1 contains a reference number for each reaction; column 2 specifies the reaction; column 3 gives an expression for the rate constant in cm^3^s^−1^ units, and column 4 gives references.

(1) In reactions 1–11, the reaction rate can be decided by the values of parameter (reduce electric field intensity) *Θ* = *E/N*, which is taken in units of 10^−16 ^V cm^2^, and in present experiment, the *Θ* is the range of 20–30 (neglect the influence by space charge);

(2) In reactions 16–26, the reaction rate constants are cited from ref. 34, where gas temperature for the reactions is 350 K;

(3) In reactions 38 and 39, M represents N_2_, O_2_, or H_2_O molecules.
